# The complete chloroplast genome sequence of *Populus simonii*, a medicinal plant for anti-lung cancer activity

**DOI:** 10.1080/23802359.2020.1788461

**Published:** 2020-07-07

**Authors:** Yi Zhuo, Jian-Bo Lin, Yin-He Yin, Fan-Cai Lai

**Affiliations:** Department of Thoracic Surgery, First Affiliated Hospital of Fujian Medical University, Fuzhou, Fujian, China

**Keywords:** *Populus simonii*, chloroplast genome, phylogenetic analysis, anti-lung cancer activity

## Abstract

Lung cancer is one of the most common malignant tumors. It is clinically divided into two types: small cell lung cancer and non-small cell lung cancer. *Populus simonii* distributed in East Asia region including China used as traditional medicine, which is an important medicinal plant for anti-lung cancer activity. The complete chloroplast genome sequence of *P. simonii* was characterized from Illumina pair-end sequencing. The chloroplast genome of *P. simonii* was 156,559 bp in length, containing a large single-copy region (LSC) of 84,825 bp, a small single-copy region (SSC) of 47,561 bp, and two inverted repeat (IR) regions of 16,494 bp. The overall GC content is 36.70%, while the corresponding values of the LSC, SSC, and IR regions are 34.5%, 30.0%, and 42.0%, respectively. The genome contains 131 complete genes, including 86 protein-coding genes (68 protein-coding gene species), 37 tRNA genes (29 tRNA species), and 8 rRNA genes (4 rRNA species). The neighbour-joining phylogenetic analysis showed that *P. simonii* and *P. qamdoensis* clustered together as sisters to other *Populus* species.

## Introduction

Lung cancer is one of the most common malignant tumors. The latter is the most common type of lung cancer pathology. Lung cancer occupies the number one cause of cancer death in the world. Although there have been some advances in the diagnosis and treatment of lung cancer, there are currently no specific treatment drugs. *Populus simonii* distributed in East Asia region including China used as traditional medicine, which is an important medicinal plant for anti-lung cancer activity, which has persisted largely in an undomesticated state that is highly resistant to different environmental stresses. Most patients with non-small cell lung cancer are not sensitive to radiotherapy and chemotherapy, so it is urgent to develop anti-cancer drugs with high efficiency and low toxicity and side effects based on the research of tumor molecular signaling pathways. Natural products are an important source of anti-tumor chemical drugs. Lung cancer refers to a disease caused by environmental and genetic factors, with dementia as the main clinical phase. Modern pharmacological studies have shown that *P. simonii* can significantly increase the number of capillary networks and accelerate blood flow, thereby restoring the function of microcirculation. At the same time, it can also reduce plasma lactic acid content and improve metabolic disorders caused by cell hypoxia. It plays an important role for anti-lung cancer activity. Studies have shown that *P. simonii* can reduce the content of lipid peroxide and erythrocyte sorbitol, increase the level of superoxide dismutase, reduce the oxidative stress reaction, and has an antioxidant effect. Studies have also found that *P. simonii* can improve the role of lung cancer. The mechanism may be: the antioxidant effect of drugs, by restoring the structure and function of nerve cells, inhibiting lipid peroxidation, improving blood rheology, and protecting against ischemia. Damaged neurons improve the pathological response of for lung cancer. Since it contains selaginellin, flavonoids and sumaflavone, it also presented anti-cancer, inhibition of immediate allergic reactions, and antihyperglycemic activities. *P. simonii* has high ecological and economic value with high levels of intraspecific genetic diversity. *P. simonii* has wide geographic distribution, high intraspecific polymorphism, adaptability to different environments, combined with a relatively small genome size. Consequently, *P. simonii* represents an excellent model for understanding how different evolutionary forces have sculpted the variation patterns in the genome during the process of population differentiation and ecological speciation (Neale and Antoine 2011). Moreover, we can develop conservation strategies easily when we understand the genetic information of *P. simonii*. In the present research, we constructed the whole chloroplast genome of *P. simonii* and understood many genome varition information about the species, which will provide beneficial help for population genetics studies of *P. simonii.*

The fresh leaves of *P. simonii* were collected from Yanzhou (116°35′E; 35°43′N). Fresh leaves were silica-dried and taken to the laboratory until DNA extraction. The voucher specimen (XXY001) was laid in the Herbarium of First Affiliated Hospital of Fujian Medical University and the extracted DNA was stored in the −80 °C refrigerator of the Key Laboratory of Department of Thoracic Surgery. We extracted total genomic DNA from 25 mg silica-gel-dried leaf using a modified CTAB method (Doyle 1987). The whole-genome sequencing was then conducted by Biodata Biotechnologies Inc. (Hefei, China) with Illumina Hiseq platform. The Illumina HiSeq 2000 platform (Illumina, San Diego, CA) was used to perform the genome sequence. We used the software MITObim 1.8 (Hahn et al. 2013) and metaSPAdes (Nurk et al. 2017) to assemble chloroplast genomes. We used *Populus qamdoensis* (GenBank: NC040868) as a reference genome. We annotated the chloroplast genome with the software DOGMA (Wyman et al. 2004), and then corrected the results using Geneious 8.0.2 (Campos et al. 2016) and Sequin 15.50 (http://www.ncbi.nlm.nih.gov/Sequin/).

The complete chloroplast genome of *P. simonii* (GenBank accession number MT482540) was characterized from Illumina pair-end sequencing. The chloroplast genome of *Populus simonii* was 156,559 bp in length, containing a large single-copy region (LSC) of 84,825 bp, a small single-copy region (SSC) of 47,561 bp, and two inverted repeat (IR) regions of 16,494 bp. The overall GC content is 36.70%, while the corresponding values of the LSC, SSC, and IR regions are 34.5%, 30.0%, and 42.0%, respectively. The genome contains 131 complete genes, including 86 protein-coding genes (68 protein-coding gene species), 37 tRNA genes (29 tRNA species) and 8 rRNA genes (4 rRNA species).

**Figure 1.**
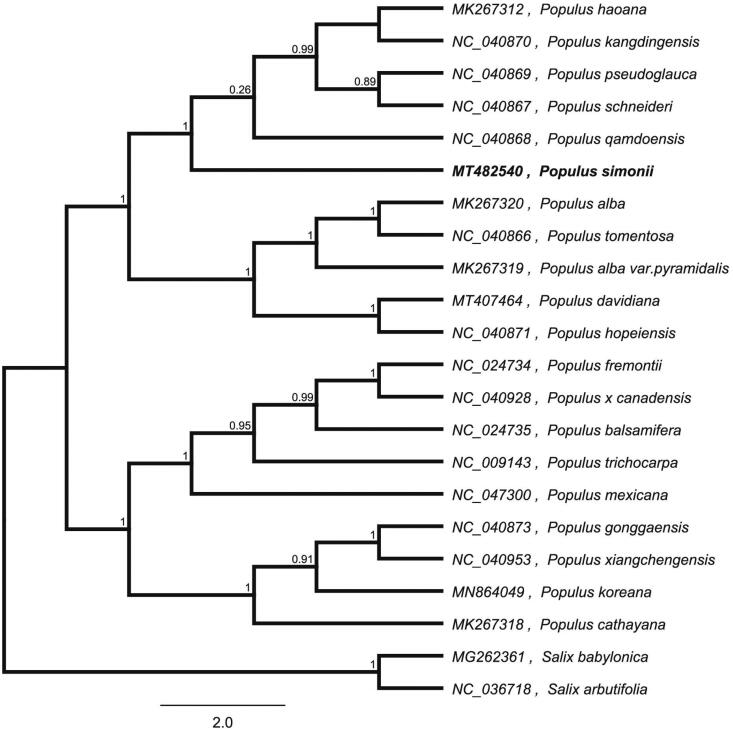
Neighbour-joining (NJ) analysis of *P. simonii* and other related species based on the complete chloroplast genome sequence.

We used the complete chloroplast genomes sequence of *P. simonii* and 21 other related species to construct phylogenetic tree. The 22 chloroplast genome sequences were aligned with MAFFT (Katoh and Standley, 2013), and then the neighbour-joining tree was constructed by MEGA 7.0 (Kumar et al. 2016). The neighbour-joining phylogenetic analysis showed that *P. simonii* and *Populus qamdoensis* clustered together as sisters to other *Populus* species (Figure 1).

## Data Availability

The data that support the findings of this study are openly available in GenBank at https://www.ncbi.nlm.nih.gov, reference number MT482540.
